# Cognitive Models of Limb Embodiment in Structurally Varying Bodies: A Theoretical Perspective

**DOI:** 10.3389/fpsyg.2021.716976

**Published:** 2021-12-23

**Authors:** Adna Bliek, Robin Bekrater-Bodmann, Philipp Beckerle

**Affiliations:** ^1^Chair of Autonomous Systems and Mechatronics, Department of Electrical Engineering, Faculty of Engineering, Friedrich-Alexander Universität Erlangen-Nürnberg, Erlangen, Germany; ^2^Department of Psychosomatic Medicine and Psychotherapy, Central Institute of Mental Health, Medical Faculty Mannheim, Heidelberg University, Mannheim, Germany

**Keywords:** bodily illusions, embodiment, structurally varying bodies, cognitive model, rubber limb illusion

## Abstract

Using the seminal rubber hand illusion and related paradigms, the last two decades unveiled the multisensory mechanisms underlying the sense of limb embodiment, that is, the cognitive integration of an artificial limb into one's body representation. Since also individuals with amputations can be induced to embody an artificial limb by multimodal sensory stimulation, it can be assumed that the involved computational mechanisms are universal and independent of the perceiver's physical integrity. This is anything but trivial, since experimentally induced embodiment has been related to the embodiment of prostheses in limb amputees, representing a crucial rehabilitative goal with clinical implications. However, until now there is no unified theoretical framework to explain limb embodiment in structurally varying bodies. In the present work, we suggest extensions of the existing Bayesian models on limb embodiment in normally-limbed persons in order to apply them to the specific situation in limb amputees lacking the limb as physical effector. We propose that adjusted weighting of included parameters of a unified modeling framework, rather than qualitatively different model structures for normally-limbed and amputated individuals, is capable of explaining embodiment in structurally varying bodies. Differences in the spatial representation of the close environment (peripersonal space) and the limb (phantom limb awareness) as well as sensorimotor learning processes associated with limb loss and the use of prostheses might be crucial modulators for embodiment of artificial limbs in individuals with limb amputation. We will discuss implications of our extended Bayesian model for basic research and clinical contexts.

## 1. Introduction

Setups such as the rubber limb illusion (RLI) (e.g., Botvinick and Cohen, 1998; Flögel et al., 2016) and related paradigms (Riemer et al., 2019) have been comprehensively used to study the embodiment of artificial limbs in normally-limbed participants. In this context, “embodiment” refers to the cognitive integration of an external object into one's body representation (Longo et al., 2008; Makin et al., 2017). In the RLI, both a participant's real but hidden limb as well as a visible artificial counterpart are touched synchronously, inducing the perception that the artificial limb belongs to the participant's body. After successful RLI induction, participants tend to locate their hidden limb closer to the rubber limb than before, a phenomenon termed “proprioceptive drift” (e.g., Botvinick and Cohen, 1998; Longo et al., 2008), which has been interpreted as proprioceptive re-calibration of the body coordinates (Botvinick and Cohen, 1998). The vividness of the RLI has been found to depend on various parameters, such as the degree of synchrony between visual and tactile stimulation (Bekrater-Bodmann et al., 2014) or visual features such as color and shape of the artificial limb (Tsakiris et al., 2010; Farmer et al., 2012). Another important factor for eliciting the RLI is whether the artificial limb is placed in the individual's limb-centered peripersonal space (PPS) (Lloyd, 2007), i.e., the intermediate surroundings of a limb, within the limits of which the integration of multimodal stimuli is facilitated (Serino, 2019). For humans, limb-centered PPS boundaries of about 30 cm for the upper limb (Lloyd, 2007) and 70 cm for the lower limb (Stone et al., 2018) have been reported.

Traditionally, the embodiment experiences elicited in the RLI have been assumed to rely exclusively on bottom-up processes (Botvinick and Cohen, 1998; Armel and Ramachandran, 2003), emphasizing a three-way interaction between vision, touch, and proprioception, which—in a connectionist tradition—leads to perceived merging of tactile and visual inputs by distortions of the position sense (Armel and Ramachandran, 2003). However, this purely bottom-up perspective is not compatible with the growing number of empirical evidence on the principles underlying limb embodiment (Litwin, 2020), as earlier described top-down modulating factors, e.g., the PPS, have been shown to influence the vividness of the RLI.

In the light of recent advances in Bayesian modeling of sensory integration (Körding and Wolpert, 2006; Körding et al., 2007; Berniker and Kording, 2011), the processes involved in the RLI have been proposed to be better modeled as multisensory combination based on probabilistic principles (Samad et al., 2015; Schürmann et al., 2019b; Litwin, 2020; Shams and Beierholm, 2021). In this view, embodiment of an external object takes place when multimodal sensory inputs are (falsely) interpreted as being caused by the same external event. Bayesian modeling has first been used by Samad et al. (2015) to predict the strength of the RLI for the hand explaining the induction of embodiment in a traditional bottom-up fashion. In their model, the induction of embodiment depends on whether a cognitive system infers common or independent causes of visual and somatosensory signals, resulting in a re-calibration of proprioceptive coordinates. The combination of sensory signals would then depend on the relative probabilities of these two posterior hypotheses derived from their prior probabilities and likelihood of sensory signals. By empirical testing of hypotheses deduced from their model, Samad et al. (2015) found strong evidence for Bayesian probabilistic processing underlying the embodiment of an artificial limb. However, a recent article by Schubert and Endres (2021) highlighted flaws in the unrealistic wide choice of the prior distributions of the model. They could not recreate realistic results using their improved prior distributions given the current model structure. Additionally, Schürmann et al. (2019b) showed that informed priors outperform the originally used uniform priors.

Crucially, the RLI can also be induced in individuals with limb amputations (e.g., Ehrsson et al., 2008) which is why this setup has been proposed to be a model for certain processes involved in the embodiment of prostheses as well. Successful embodiment of a prosthesis is important as the amputation of a limb severely disrupts a person's physical integrity. There are preliminary reports that most individuals with amputations can achieve embodiment of their prosthesis (Bekrater-Bodmann, 2020) the processes of which have been related to positive clinical outcomes (e.g., Imaizumi et al., 2016; Bekrater-Bodmann, 2021; Bekrater-Bodmann et al., 2021). The psychometric structure behind experimentally-induced short-termed RLI experiences in normally-limbed participants (Longo et al., 2008) and real-life long-termed prosthesis embodiment in limb amputees (Bekrater-Bodmann, 2020) show substantial qualitative similarity, which is remarkable, given the striking differences in the participant's physical integrity. Although there is reason to assume that differential neurocognitive mechanisms contribute to embodiment experiences in the RLI and prosthesis use, with the latter probably relying on long-term sensorimotor learning rather than short-term multimodal sensory combination (cf., Zbinden and Ortiz-Catalan, 2021), the psychometric similarities suggest at least partly overlapping, potentially Bayesian processes.

However, the question arises how bodily self-experiences in general and prosthesis embodiment in particular can be theoretically explained in a unified fashion taking into account and improving on the currently used Bayesian modeling approaches. A unified modeling framework could be a step toward prediction of factors improving embodiment of artificial limbs and could thus improve user experience. The authors of the present article propose a 2-fold extension of the current modeling approaches in accordance with the upper two levels of cognitive modeling proposed by Marr (1982), which has been proposed in earlier research to describe the different underlying task of modeling approaches (e.g., Schürmann and Beckerle, 2020; Shams and Beierholm, 2021). Firstly, starting on the computational theory level, we propose to improve the current model structure, and extend the models for structurally varying bodies taking into account individual differences in perception of embodiment. Secondly, on the algorithmic level, we propose to incorporate top-down modulating factors in the priors of the cognitive models, according to Litwin (2020).

## 2. Limb Embodiment in Structurally Varying Bodies

Normally-limbed and amputated individuals differ in important representational and perceptual characteristics, which have to be considered when cognitive modeling is applied to the processes underlying artificial limb embodiment. Thus, limb amputees often report the presence of a phantom limb (Kooijman et al., 2000), i.e., the persistent perception of a body part that has been removed. The proprioceptive presence of a phantom limb can be made use of in the induction of embodiment: in some individuals with amputations, tactile stimulation applied to the residual limb can trigger a touch sensation in the phantom limb, known as “referred sensations”, which might be a consequence of neuroplastic changes in the somatotopic body maps in the brain (Ramachandran et al., 1992). If the location of the elicited sensations in the phantom corresponds to the visual location of touch applied to the artificial limb, embodiment experiences can be facilitated (Ehrsson et al., 2008). Furthermore, there is preliminary evidence that prostheses interact with the phantom limb in terms of perceptual co-location (the phantom “occupies” the space of the prosthesis; Giummarra et al., 2008) which might also foster the embodiment of the prosthetic device. Postural phantom limb disturbances, however, could interfere with the incorporation of the artificial limb and consequently reduce embodiment (cf., Foell et al., 2014).

Moreover, limb amputation is associated with a shrinkage in the extent of PPS representation, with a shift of its boundaries toward the stump (Canzoneri et al., 2013), which might explain why prosthesis embodiment is strong for long residual limbs and low for short ones (Bekrater-Bodmann, 2020): in short residual limbs, the prosthesis might “stick out” of the PPS boundaries which interferes with its embodiment (cf., Lloyd, 2007). Whether or not phantom limb perceptions are associated with normal PPS extent, however, remains unknown.

## 3. Models Predicting Embodiment for Structurally Varying Bodies

Given both the perceptual similarities and potential mechanistic differences, i.e., integration of multimodal sensory input vs. sensorimotor learning processes, between short-term and long-term embodiment in normally-limbed and amputated individuals, the combination of Bayesian and connectionist models and the modulation of priors seem promising for the prediction of experiences in both groups. However, it is currently unclear how these models should be combined or adapted. Current embodiment models do not cover structural body varieties, e.g., limb presence or absence, since priors do not take into account inter-individual differences in body representation, e.g., differences in PPS extent and different underlying mechanisms. One crucial issue could relate to quantitatively different weighting of certain sensorimotor factors in normally-limbed vs. amputated individuals, while the structure of the model itself remains unaffected. This might allow for the integration of different PPS representations in amputated and normally-limbed bodies, as preliminary indicated by Canzoneri et al. (2013). Samad et al. (2015) highlighted that their proposed framework is extendable to incorporate such additional variables by adding individual prior knowledge, e.g., by adding tactile, proprioceptive, and visual priors and adapting their sensitivity to the individual or a group of people. The importance of prior knowledge is highlighted by recent evidence suggesting that the prediction quality for behavioral correlates of the RLI can be enhanced by entering informed priors to the probabilistic model (Schürmann et al., 2019b). Litwin (2020) further opts for the inclusion of individual dispersions of coupling priors for modeling the potentially important, but largely neglected, top-down effects. Thus, beyond bottom-up processes, the human cognitive system seems to use prior knowledge of cross-modal correlations, e.g., the correlation between visual and tactile stimulation, to modulate sensory integration in PPS (Parise et al., 2012, 2013), which might be subject to individual sensorimotor experiences and learning. The embodiment of an artificial limb has been linked to remapping of PPS boundaries to the location of the artificial limb (Brozzoli et al., 2012), suggesting that its representation is shaped by top-down influences.

**Figure 1:**
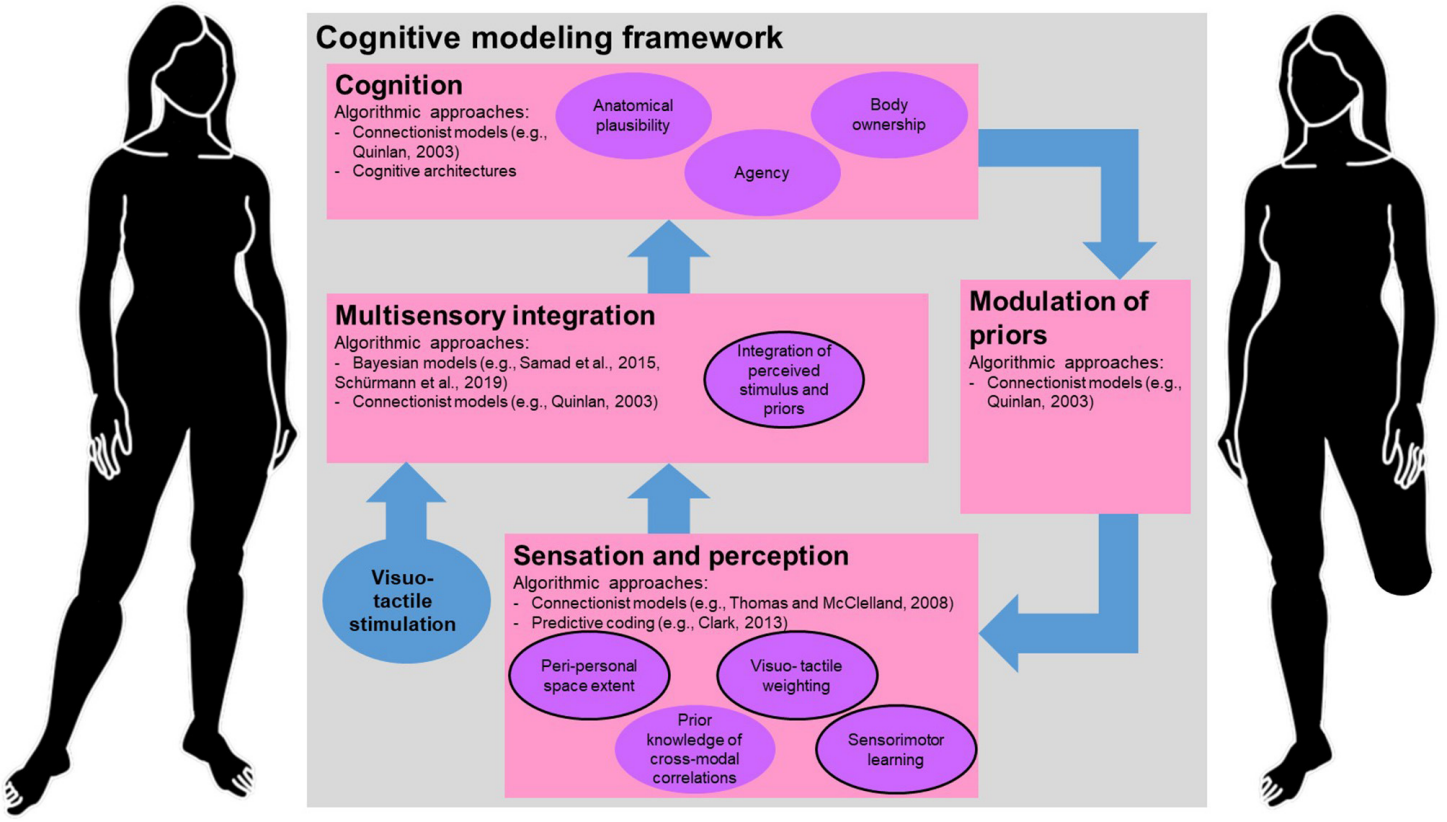
The proposed cognitive modeling framework suggests a conceptual structure representing embodiment mechanisms on the computational level. Submodels (pink boxes) are connected by information flows (blue arrows). The submodels contain suggestions for algorithmic approaches to realize the functions represented in the purple ellipses. Among those, mechanisms that appear important for long-term differences in embodiment, i.e., sensorimotor learning in individuals with amputations, are outlined. The framework further suggests how to model the integration of visuo-tactile with proprioceptive information. Bottom-up information is used as input for the model of cognition from the perceived stimulation as well as from prior knowledge. The prior knowledge is modulated using top-down information from earlier experiences.

To factor in individual representational differences, we suggest to improve the existing Bayesian models with respect to estimation accuracy, specifications for individual users, and online capabilities. Figure 1 provides a conceptual perspective of an extended framework to include individual differences. Starting from multisensory models aiming to predict the perceived limb location, i.e., the proprioceptive drift (Samad et al., 2015; Schürmann et al., 2019b), extending the models with sensorimotor information in order to cover more complex behavioral and psychological outcomes. To this end, we suggest extending the current Bayesian model considering the upper two levels of Marr (1982): the computational theory level, describing what a system is doing and what functions are needed to complete this goal, and the algorithmic level, outlining how the system could be implemented (Marr, 1982; Dennett, 1987).

The goal of the proposed framework is to estimate the embodiment of an artificial limb for an individual taking into account structural differences of their bodies. We suggest that this goal can be realized by combining established models of multisensory integration with models of perception and higher cognition, and extending the overall framework by experience-modulated priors. These changes are indicated in the addition of the model of cognition, the models of sensation and perception, and the top-down modulation in Figure 1. The added priors would not be individualized but represent general influences of experience, i.e., irrespective of structural body variations. Computationally, this could be covered by a top-down modulation to predict influences of previous experiences on prior couplings, e.g., visuo-tactile integration or sensorimotor learning, using the implementation of learning-based models of inter- and intramodal sensory signals (Van Dam et al., 2014; Parise, 2016; Noel et al., 2018; Litwin, 2020; Press et al., 2020).

On the algorithmic level, we propose to extend the approaches and mechanisms in the submodels of sensation and perception, cognition, and top-down modulation, see algorithmic approaches in Figure 1. The model of cognition adds psychometric measures of embodiment, e.g., perceived agency and body ownership, in order to include individual perceptual outcomes in addition to the proprioceptive measures. To include more individualized information in the model of sensation and perception, Bayesian and connectionist methods as well as predictive coding are promising for the perceptual submodels, e.g., by adding sensorimotor learning, (Thomas and McClelland, 2008; Clark, 2013; Samad et al., 2015; Schürmann et al., 2019a,b). The top-down connection between the model of cognition, and the model of sensation and perception is adding experience-modulated priors (cf., Ingram et al., 2017), incorporating recent evidence for top-down modulation of adaptive sensory representations in the brain (Makino et al., 2016). We propose adding a top-down modulation of priors to incorporate information about individual PPS, visuo-tactile weighting, sensorimotor learning and prior knowledge of cross-modal correlations, indicated by the ellipses in the submodel of sensation and perception in Figure 1. This pathway could be realized in a connectionist fashion, e.g., by the implementation of artificial neural networks (Quinlan, 2003; Zhong, 2015). Artificial neural networks, as well as network architecture in the brain (Graham, 1982), use feedback information to update the weights of the connections between neurons. This process makes them adaptable to individual differences, while also modeling processes that are valid on group level. These approaches appear to be particularly promising for limb amputees who are characterized by high variability in sensorimotor experiences related to the use of prostheses.

To ensure accurate models for structurally varying bodies, the suggested algorithmic model adaptations should be performed iteratively using human-in-the-loop experiments with individuals with structurally varying bodies, e.g., people with/without amputation, to verify and adapt the implemented models and priors. We postulate that the overall cognitive modeling framework should be generally applicable to structurally varying bodies at computational level. The methods selected on algorithmic level might be identical, but should vary in the parameterization that represents individual effects, e.g., artificial neural network weights.

## 4. Conclusion

Both the similarities and the differences of limb embodiment in individuals with structurally varying bodies show a need for an extension of currently used cognitive models for normally-limbed people. These models should be adapted to consider individual limb differences by incorporating further parameters such as the peripersonal space and adapting the weighting of included parameters iteratively to the individual. Such extensions could not only help to explain and predict embodiment of prostheses but also highlight individual factors that facilitate or hinder embodiment of rehabilitative devices in general.

The current research points toward prior sensorimotor experiences and the peripersonal space extent taking influence on the embodiment of (artificial) limbs. Thus, we advocate to create a cognitive modeling framework that extends current approaches with top-down modulations to represent individual structural and other representational differences and make algorithmic suggestions to realize its implementation, e.g., using artificial neural networks or cognitive architectures.

Furthermore, modeling embodiment for both individuals with and without amputation will enable the characterization of the variability (or invariability) of different parameters of the model, e.g., the sensitivity of priors or the importance of used prior knowledge in cognitive architectures or artificial neural networks. In other words, the comparison of the models' dynamics for structurally varying bodies will reveal to which degree the bodily self is subject to plastic adaptions in response to structural alterations of the physical body. To accurately model the variability in the processes involved in limb embodiment, experiments with participants with and without amputations will be needed before adapting the models to inform theoretical considerations. Supported by neuropsychological research, the proposed modeling approaches might foster our understanding of the mechanisms underlying limb embodiment and the predictive power of cognitive models, which might in turn be used to improve the design and control of assistive devices.

## Author Contributions

All authors listed have made a substantial, direct, and intellectual contribution to the work and approved it for publication.

## Funding

This work has been supported by the Deutsche Forschungsgemeinschaft (DFG; BE 5723/4-1) and the Volkswagen-Foundation (Az. 9B 007).

## Conflict of Interest

The authors declare that the research was conducted in the absence of any commercial or financial relationships that could be construed as a potential conflict of interest.

## Publisher's Note

All claims expressed in this article are solely those of the authors and do not necessarily represent those of their affiliated organizations, or those of the publisher, the editors and the reviewers. Any product that may be evaluated in this article, or claim that may be made by its manufacturer, is not guaranteed or endorsed by the publisher.
